# Systemic air embolism after percutaneous computed tomography-guided lung biopsy due to a kink in the coaxial biopsy system: a case report

**DOI:** 10.1186/s12880-018-0245-9

**Published:** 2018-01-27

**Authors:** Hsu-Chao Chang, Mei-Chen Yang

**Affiliations:** 1 Department of Radiology, Taipei Tzu-Chi Hospital, Buddhist Tzu-Chi Medical Foundation, No. 289, Jianguo Rd, Xindian Dist, New Taipei City, 23143 Taiwan; 2 School of Medicine, Tzu Chi University, Hualien, Taiwan; 3 Division of Pulmonary Medicine, Department of Internal Medicine, Taipei Tzu-Chi Hospital, Buddhist Tzu-Chi Medical Foundation, No. 289, Jianguo Rd, Xindian Dist, New Taipei City, 23143 Taiwan

**Keywords:** Complication, Lung mass, Chest imaging

## Abstract

**Background:**

Systemic air embolism is a rare but potentially life-threatening complication of percutaneous computed tomography (CT)-guided lung biopsy. The incidence might be underestimated because of failure to diagnose this adverse event in asymptomatic patients; early recognition is difficult.

**Case presentation:**

We report the case of a 73-year-old man with systemic air embolism, a complication of percutaneous CT-guided lung biopsy, due to a kink in the coaxial biopsy system. Serial post-procedure CT scans demonstrated the causal relationship.

**Conclusions:**

Sequential post-biopsy CT scans demonstrated a causal relationship between this systemic air embolism and percutaneous biopsy, and allowed the radiologist to track the course of the emboli and their resolution. Awareness of air entry via the introducer needle and an early post-biopsy CT scan are crucial for early detection of systemic air embolism. If air embolism occurs in an asymptomatic patient, we recommend performing a delayed chest CT scan to follow the air’s course.

## Background

Systemic air embolism after percutaneous computed tomography (CT)-guided lung biopsy is very rare. The estimated incidence is 0.061–0.21% [1, 2], but could be underestimated because of failure to diagnose this adverse event in asymptomatic patients [3]. For example, in a study published in 2012, Freund et al. found that the incidence of radiologically proven systemic air embolism was 3.8%, whereas the incidence of clinically apparent systemic air embolism was 0.49% [4]. Thus, the incidence of post-biopsy systemic air embolism might be higher than is estimated, and sequential CT scans of the entire thorax instead of only the biopsy area might be worth performing to provide a definitive diagnosis of systemic air embolism [5]. Generally, systemic air embolism is thought to occur via two mechanisms. First, if the tip of the biopsy needle is lodged in a pulmonary vein and the inner stylet is removed, air embolism occurs during rapid inspiration when atmospheric pressure exceeds pulmonary venous pressure. Second, when the needle simultaneously traverses an air-containing space and adjacent pulmonary vein, a fistula can occur, resulting in air entering the pulmonary vein when the alveolar pressure is greater than the pulmonary venous pressure [6]. Air can enter via a fistula tract, connecting an air-containing space to a pulmonary vein when alveolar pressure is high, for example, during coughing. To prevent air embolism, the introducer needle should always be occluded by the inner stylet, saline drops, or the operator’s finger. Moreover, the patient should be instructed to avoid breathing deeply or coughing forcefully during the procedure [7, 8]. It is believed that, following lung biopsy and needle withdrawal, a large number of alveolar-venous or bronchial-venous fistulas can develop along the needle’s trajectory. During vascular injury, the walls of small vessels are usually retracted; spontaneous adhesion occurs, forming a seal. When a patient exhibits any symptoms suggestive of air embolism during the lung biopsy procedure, the needle should immediately be removed [8, 9]. The clinical manifestations of air embolism vary, depending on the exact location of the arterial embolus and the volume of air disseminated into the vessels. Once air enters the systemic circulation, it will enter the respective arterial end beds [1, 8]. Functional end arteries can be occluded by even a small volume of air. Only 2 mL of air injected directly into the cerebral circulation is enough to be fatal, and 0.5–1.0 mL of air injected into a coronary artery can lead to cardiac arrest [10]. Initial management includes immediate administration of 100% oxygen and placing the patient in a slight Trendelenburg position [7]. Early hyperbaric oxygen therapy is recommended for patients with cerebral air embolism [5].

Previously reported cases have only demonstrated imaging findings as existing air emboli in post-procedure CT scans. Imaging findings at different times, and the use of those findings to track the course and resolution of air emboli, have not been described. We report a case of systemic air embolism after CT-guided biopsy due to a kink in the coaxial biopsy system, and show the course followed by the emboli, demonstrated on sequential CT scans.

## Case presentation

A 73-year-old man with pneumoconiosis, chronic obstructive pulmonary disease, and hypertension who had previously had a cerebrovascular accident and had undergone surgery for gastric cancer ten years earlier, was brought to our emergency department with a one-day history of acute-onset tremor and leg weakness. The chest radiograph and chest CT scan showed a large mass in the right middle lobe (RML) that was out of keeping with his pneumoconiosis. Lung malignancy was of concern; hence, a CT-guided percutaneous lung biopsy was suggested and was performed after obtaining informed consent.

**Fig. 1:**
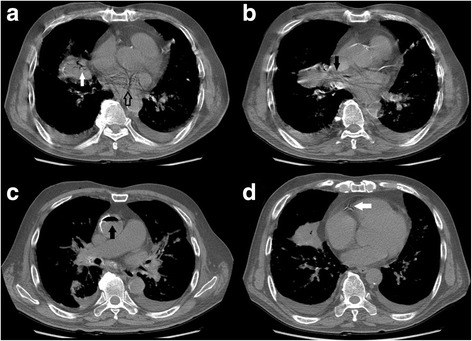
Series of computed tomography scans demonstrating the movement of the air emboli. Immediate post-procedure computed tomography scans demonstrated free air with flow-related motion artifact in **a** the right middle lobe mass (white arrow), and the left atrium (open arrow), and **b** the right pulmonary vein (black arrow). After 50 s, a follow-up computed tomography scan demonstrated air collections in **c** the ascending aorta (black arrow) and **d** right coronary artery (white arrow)

**Fig. 2:**
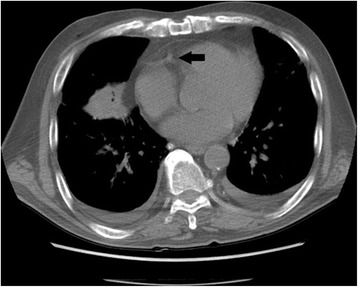
Delayed chest computed tomography scan, performed after 10 min, demonstrating total resolution of the air embolus in the right coronary artery

A coaxial biopsy system with a 19-gauge introducer needle and a 20-gauge core biopsy needle (length, 17 cm; Temno, CareFusion, France) was selected for this procedure. Once the introducer needle had reached the lesion, the internal stylet was removed and the core biopsy needle was inserted to procure a specimen. However, after obtaining the first specimen, we were unable to withdraw the core biopsy needle because of a kink. Several attempts to withdraw it resulted in it being pushed forward and kinked again; finally, it was forcibly removed. The core biopsy needle was found to be angulated proximally. Thereupon, free air sprang up from the introducer needle. Hence, the stylet was reinserted and the introducer needle was immediately removed. After withdrawal of the instrument, the patient coughed forcefully. An immediate post-biopsy CT scan was performed; it demonstrated free air with flow-related motion artifact within the RML mass, the left atrium, and the right pulmonary vein (Fig. 1a and b). A follow-up CT scan of the entire thorax demonstrated air collections in the ascending aorta and the right coronary artery (Fig. 1c and d). Because the patient had no symptoms or signs of acute stroke or myocardial infarction, he was kept in the CT room and oxygen was administered. Ten minutes later, a delayed chest CT scan demonstrated no air collections in the aorta or left heart, and total resolution of the air embolus in the right coronary artery (Fig. 2). The patient’s condition was stable and an electrocardiogram showed no ST-segment elevation. He was thus transferred to the intensive care unit for close monitoring.

During his stay in the intensive care unit, neither air embolism nor new neurologic deficits developed, and his troponin I and CK-MB levels were normal. Five days later, brain magnetic resonance imaging was performed; it showed no evidence of acute infarction. The patient was transferred to the ward and was discharged days later in a stable condition. Table 1 shows the timeline of course of events.

**Table 1:** Timeline of events

Date	Event
6 Nov	Admission for CT-guided lung biopsy
7 Nov	Systemic air embolism after percutaneous CT-guided lung biopsy due to a kink in the coaxial biopsy system
7 Nov	No ST-segment elevation on electrocardiogram; normal troponin I and CK-MB levels
12 Nov	No acute infarct on brain magnetic resonance imaging
17 Nov	Discharged in a stable condition

## Discussion

As demonstrated in this report, post-biopsy sequential CT scans can show the causal relationship between systemic air embolism and percutaneous biopsy, and can track the course and resolution of such emboli. The serial CT scans performed on our patient showed air with flow-related motion artifact in the lung mass, pulmonary vein, left heart, and then in the aorta and coronary artery. Later, the CT scans showed resolution of the air collections in the aorta and left heart, and almost total resolution of the air embolus in the coronary artery. Such evolution occurred quickly. Had the radiologists been unaware of this complication, CT scans would have been delayed and the systemic air emboli would either not have been detected or the diagnosis would have been delayed.

**Fig. 3:**
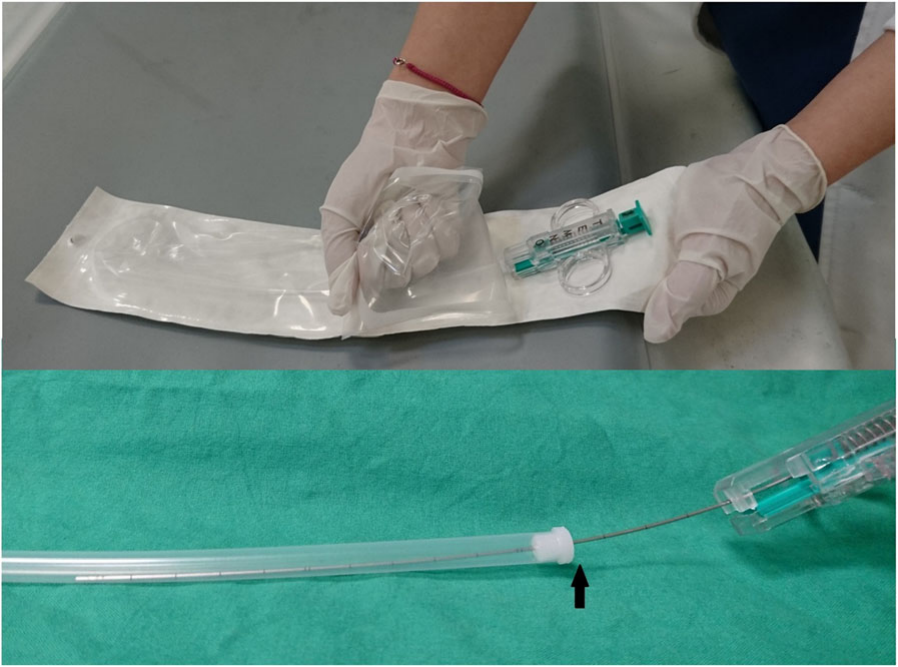
Inappropriate removal of the core biopsy needle from its packaging. The upper image demonstrates an inappropriate method of removing the core biopsy needle from its packaging, resulting in angulation at the fulcrum where pressure was applied (lower image, black arrow)

How can one avoid causing an angulation deformity in the core biopsy needle, thereby causing it to kink and get stuck in the introducer needle? Although blood clots can cause the biopsy needle to adhere to the introducer needle, especially when a long instrument is used, such adherence can be managed by flushing the introducer needle. After several experiments, we determined that the core biopsy needle deformity had occurred because the needle had been inappropriately removed from its packaging, resulting in angulation at the fulcrum where pressure was applied (Fig. 3). If the needle is inadvertently kinked, the biopsy set should be instantly discarded to prevent such severe complications as occurred in this case.

In the case presented, the air definitely came from the atmosphere because we witnessed air being drawn into the introducer needle. Moreover, the to-and-fro motion of the kinked core biopsy needle in the introducer needle may have evacuated the mass. Delayed reinsertion of the introducer needle’s stylet after forcibly removing the kinked biopsy needle also played an important role. Early awareness of air being drawn into the introducer needle is very important. An immediate series of CT scans to trace the air’s course is crucial for early detection of systemic air embolism.

Initial management includes immediate administration of 100% oxygen, and placing the patient in a slight Trendelenburg position [7]. Early hyperbaric oxygen therapy is recommended for patients with cerebral air embolism [5]. However, there is no standard protocol for managing asymptomatic patients; we recommend performing delayed post-procedure CT scans to evaluate the air’s course.

Two major events need to be considered: Embolic stroke and myocardial infarction. Clinical features that support the diagnosis of cardioembolic stroke include the sudden onset of neurological deficits, an interval from onset to maximal deficit of < 5 min, and rapid regression of symptoms [11]. The symptoms of cerebral arterial air embolism develop suddenly. The clinical presentation is determined by the absolute amount of air and the intracranial vessel territories affected by the embolic event [12]. If delayed post-procedure CT scans show no more air in the left heart or in the thoracic aorta, there is no further concern of cerebral air embolism occurring. However, the symptoms of myocardial infarction may be atypical and silent [13, 14]. The electrocardiogram demonstrates ST-segment elevation rapidly (within 30 s) after manual transient occlusion of a coronary artery [15]. The incidence of acute non-ST elevation myocardial infarction may be higher than acute ST elevation myocardial infarction [16]. In our daily practice, a post-biopsy CT scan is performed routinely in every patient to evaluate complications. If air embolism is detected in a routine post-biopsy CT scan, another delayed CT scan is recommended to follow the air’s course in an asymptomatic patient. If this delayed CT scan does not demonstrate air emboli in the coronary artery, left heart, or thoracic aorta, either no air entry occurred or the coronary artery has recanalized. In this patient, we evaluated the coronary arteries in the delayed post-procedure CT scan images to ensure that the air embolism had completely resolved. However, if the air embolism persists or progresses, the patient should be intensively monitored (e.g. symptom and electrocardiogram monitoring, and serial measurement of cardiac enzymes).

## Conclusion

Sequential post-biopsy CT scans of this patient demonstrated the causal relationship between systemic air embolism and percutaneous biopsy, and allowed the radiologist to track the course and resolution of the emboli. Awareness of air entry via the introducer needle and an early post-biopsy CT scan are crucial for early detection of systemic air embolism. We recommend that a delayed chest CT scans be performed to follow the air’s course when air embolism is detected in an asymptomatic patient.

## Abbreviations

CT: Computed tomography
RML: Right middle lobe
